# Mass Media Use to Learn About COVID-19 and the Non-intention to Be Vaccinated Against COVID-19 in Latin America and Caribbean Countries

**DOI:** 10.3389/fmed.2022.877764

**Published:** 2022-06-13

**Authors:** Guido Bendezu-Quispe, Jerry K. Benites-Meza, Diego Urrunaga-Pastor, Percy Herrera-Añazco, Angela Uyen-Cateriano, Alfonso J. Rodriguez-Morales, Carlos J. Toro-Huamanchumo, Adrian V. Hernandez, Vicente A. Benites-Zapata

**Affiliations:** ^1^Escuela de Medicina, Universidad César Vallejo, Trujillo, Peru; ^2^Red Internacional en Salud Colectiva y Salud Intercultural, Mexico, Mexico; ^3^Sociedad Científica de Estudiantes de Medicina de la Universidad Nacional de Trujillo, Trujillo, Peru; ^4^Grupo Peruano de Investigación Epidemiológica, Unidad de Investigación para la Generación y Síntesis de Evidencias en Salud, Universidad San Ignacio de Loyola, Lima, Peru; ^5^Universidad Científica del Sur, Lima, Peru; ^6^Escuela de enfermería, Universidad Privada San Juan Bautista, Lima, Peru; ^7^Instituto de Evaluación de Tecnologías Sanitarias en Salud e Investigación–IETSI, EsSalud, Lima, Peru; ^8^Medecins Sans Frontieres, Health Politics Team, Brussels, Belgium; ^9^Latin American Network of COVID-19 Research (LANCOVID), Pereira, Colombia; ^10^Grupo de Investigación Biomedicina, Faculty of Medicine, Fundación Universitaria Autónoma de las Americas, Pereira, Colombia; ^11^Unidad de Investigación para la Generación y Síntesis de Evidencias en Salud, Vicerrectorado de Investigación, Universidad San Ignacio de Loyola, Lima, Peru; ^12^Unidad de Investigación Multidisciplinaria, Clínica Avendaño, Lima, Peru; ^13^Health Outcomes, Policy, and Evidence Synthesis (HOPES) Group, University of Connecticut School of Pharmacy, Storrs, CT, United States; ^14^Unidad de Revisiones Sistemáticas y Metaanálisis, Guías de Práctica Clínica y Evaluaciones Tecnológicas Sanitarias, Universidad San Ignacio de Loyola, Lima, Peru

**Keywords:** mass media, SARS-CoV-2, COVID-19, Latin America, vaccines

## Abstract

**Background:**

The Latin American and Caribbean (LAC) region has been one of the regions most affected by the COVID-19 pandemic, with countries presenting some of the highest numbers of cases and deaths from this disease in the world. Despite this, vaccination intention is not homogeneous in the region, and no study has evaluated the influence of the mass media on vaccination intention. The objective of this study was to evaluate the association between the use of mass media to learn about COVID-19 and the non-intention of vaccination against COVID-19 in LAC countries.

**Methods:**

An analysis of secondary data from a Massachusetts Institute of Technology (MIT) survey was conducted in collaboration with Facebook on people's beliefs, behaviors, and norms regarding COVID-19. Crude and adjusted prevalence ratios (aPR) with their respective 95% confidence intervals (95%CI) were calculated to evaluate the association between the use of mass media and non-vaccination intention using generalized linear models of the Poisson family with logarithmic link.

**Results:**

A total of 350,322 Facebook users over the age of 18 from LAC countries were included. 50.0% were men, 28.4% were between 18 and 30 years old, 41.4% had a high school education level, 86.1% lived in the city and 34.4% reported good health condition. The prevalence of using the mass media to learn about COVID-19 was mostly through mixed media (65.8%). The non-intention of vaccination was 10.8%. A higher prevalence of not intending to be vaccinated against COVID-19 was found in those who used traditional media (aPR = 1.36; 95%CI: 1.29–1.44; *p* < 0.001) and digital media (aPR = 1.70; 95%CI: 1.24–2.33; *p* = 0.003) compared to those using mixed media.

**Conclusion:**

We found an association between the type of mass media used to learn about COVID-19 and the non-intention of vaccination. The use of only traditional or digital information sources were associated with a higher probability of non-intention to vaccinate compared to the use of both sources.

## Introduction

Since the WHO declared the COVID-19 pandemic in March 2020 (1), it is estimated that as of December 20, 2021, there were more than 274,000 cases globally and more than five million deaths from this disease (2). The vaccine against COVID-19 is the most cost-effective strategy to combat this pandemic, and it is estimated that as of December 20, 2021, more than eight billion doses of the vaccine have been administered worldwide.

To date, several highly effective vaccines have been licensed to reduce the incidence of hospitalization and death. However, vaccine coverage remains insufficient (3, 4) due to aspects such as low acceptance of vaccination (5) as in the Middle East/North Africa, Europe and Central Asia, and West/Central Africa, which reported higher proportions of COVID-19 vaccine hesitancy (6). This implies a public health problem since the control of an infectious disease through the use of vaccines involves having high vaccination coverage, which in the case of COVID-19 has been suggested should be of 70 to 80% of the population (7).

The Strategic Advisory Group of Experts on Immunization (SAGE) of the World Health Organization (WHO) defines vaccine reluctance as a “delay in accepting the vaccine or refusing the vaccine despite its availability” (8). This phenomenon is influenced by complacency, trust and convenience (5) and is considered by the WHO as one of the 10 greatest challenges of global health (9).

Different factors influence vaccine reluctance, including the information disseminated by the mass media (10). Although these media played an important role in disseminating community mitigation strategies and other favorable measures during the pandemic (11), their impact is not always positive. Various studies have shown that media coverage of coronavirus news during geo-blockades, prolonged quarantines, and financial and social hardships induced fear and provoked psychological stress (11). Likewise, they fueled rumors, hoaxes, and misinformation about the etiology, the results, the prevention, and the cure of the disease (12). In this sense, the mass media plays a key role in the perception of vaccines (13–15). Indeed, before the pandemic, critical digital media against vaccines influenced vaccination intentions (16), suggesting the importance of designing public policies to counteract this influence.

Latin America and the Caribbean (LAC) is one of the regions most affected by the pandemic (17), and the need to prioritize access to vaccines against COVID-19 (18) has been called for in low- and middle-income countries. Countries such as Mexico, Brazil, and Peru are among those with the highest number of cases and deaths from this disease in the world (2). Although previous studies have described a high vaccination intention in the region, this is not homogeneous (10, 19), and some access barriers have hindered the early and extensive vaccination campaign against COVID-19 in the region (20). Likewise, it has been described that in LAC there is greater mistrust of science so that whoever provides information on vaccines, beyond medical or scientific authorities, maybe more persuasive (21). This is relevant because the population with low acceptance of the vaccine could respond positively to available and accessible information from promoters related to the general population (22–24). Although some studies in Latin American countries have evaluated some aspects of the impact of the mass media and social networks on the search for information during the pandemic (25, 26), to the best of our knowledge, no study has evaluated the influence of the mass media related to the intention of vaccination. Therefore, the objective of our research was to evaluate the association between the use of mass media news and information to learn about COVID-19 and the non-intention of vaccination against COVID-19 in LAC countries.

## Methods

### Study Design

A secondary analysis was performed using a database compiled by the Massachusetts Institute of Technology (MIT) in collaboration with Facebook. This survey aimed to assess beliefs, behaviors, and norms due to COVID-19. Data collection began on July 7, 2020, and ended on March 28, 2021. It was conducted in more than 60 countries and translated into 51 languages. Two versions of the survey were available. First, in countries with a sufficient pool of users to sample, a multi-wave survey was conducted continuously over several 2-week waves with the goal of collecting 3,000 respondents per wave. Second, in countries with a limited survey pool, we fielded a snapshot survey in which Facebook aimed to deliver 3,000 respondents over a 2-week period.

### Population, Sampling, and Sample

The survey included participants aged 18 or older who were Facebook users. Participants who resided in LAC and who participated in the survey between July 7, 2020, and March 28, 2021, were included. Participants were asked the question: In the past week, did you see more or less news than you wanted to see about coronavirus (COVID-19)? to which the answers could be: (1) Much less, (2) Less, (3) About the right amount, (4) More, or (5) Much more. However, only those who answered (i) About the right amount, (ii) More, and (iii) Much more were considered. Finally, participants who did not present data for the variables of interest and did not have the weighting factor to perform the corresponding analyses were excluded.

### Variables

The outcome variable was the non-intention to be vaccinated, which was operationalized from the answer to the question: If a vaccine for COVID-19 becomes available, would you choose to get vaccinated? The possible answers to this question were: yes, no, I don't know, I have already been vaccinated. The construction of the variable was carried out considering only those who answered yes or no.

The exposure variable was defined using the question: In the past week, from which of the following, if any, have you received news and information about COVID-19? with the following response alternatives: (1) Online sources (websites, apps, social media), (2) Messaging apps/SMS/text messaging, (3) Newspapers, (4) Television and (5) Radio. For the analysis, three categories of exposure to information media were constructed: digital media (online sources or messaging apps), traditional media (newspapers or television or radio) and mixed media (digital media and traditional media).

Other variables included were gender (male, female, non-binary), age (18–30, 31–40, 41–50, 51–60, 61–70, 71–80, over 80), educational level (less than primary school, primary school, secondary school, college/university, graduate school), area of residence (city, town, village or rural area) and state of health (poor, fair, good, very good, excellent).

### Statistical Analysis

The databases were compiled and downloaded in “.txt” text format, then imported into the statistical package STATA v15.0 (StataCorp, TX, USA). All analyses were performed considering the complex sampling of the survey using the svy command.

A descriptive analysis was performed using absolute frequencies and weighted proportions with their respective 95% confidence intervals (95% CI) according to the complex sampling of the survey. In the bivariate analysis, we used the Pearson Chi-square test with Rao-Scott correction. To evaluate the association between the mass media use to learn about COVID-19 and the non-intention to be vaccinated, generalized linear models of the Poisson family with log link function were constructed. The crude prevalence ratio (cPR) and adjusted prevalence ratio (aPR) were calculated with their respective 95% CI for the associations studied. Adjustment for confounders was performed considering an epidemiological approach. Collinearity was evaluated using variance inflation factors (VIF), considering a cut-off point <10. A *p* < 0.05 was considered statistically significant for all analyses performed.

### Ethical Aspects

Before starting the survey, all participants provided informed consent. An analysis of a secondary database that did not have personal identifiers and that respected the integrity of the participants was performed. Access to the data was granted by the Massachusetts Institute of Technology, Boston, United States of America.

## Results

### Selection of the Sample Included in the Study

**Figure 1:**
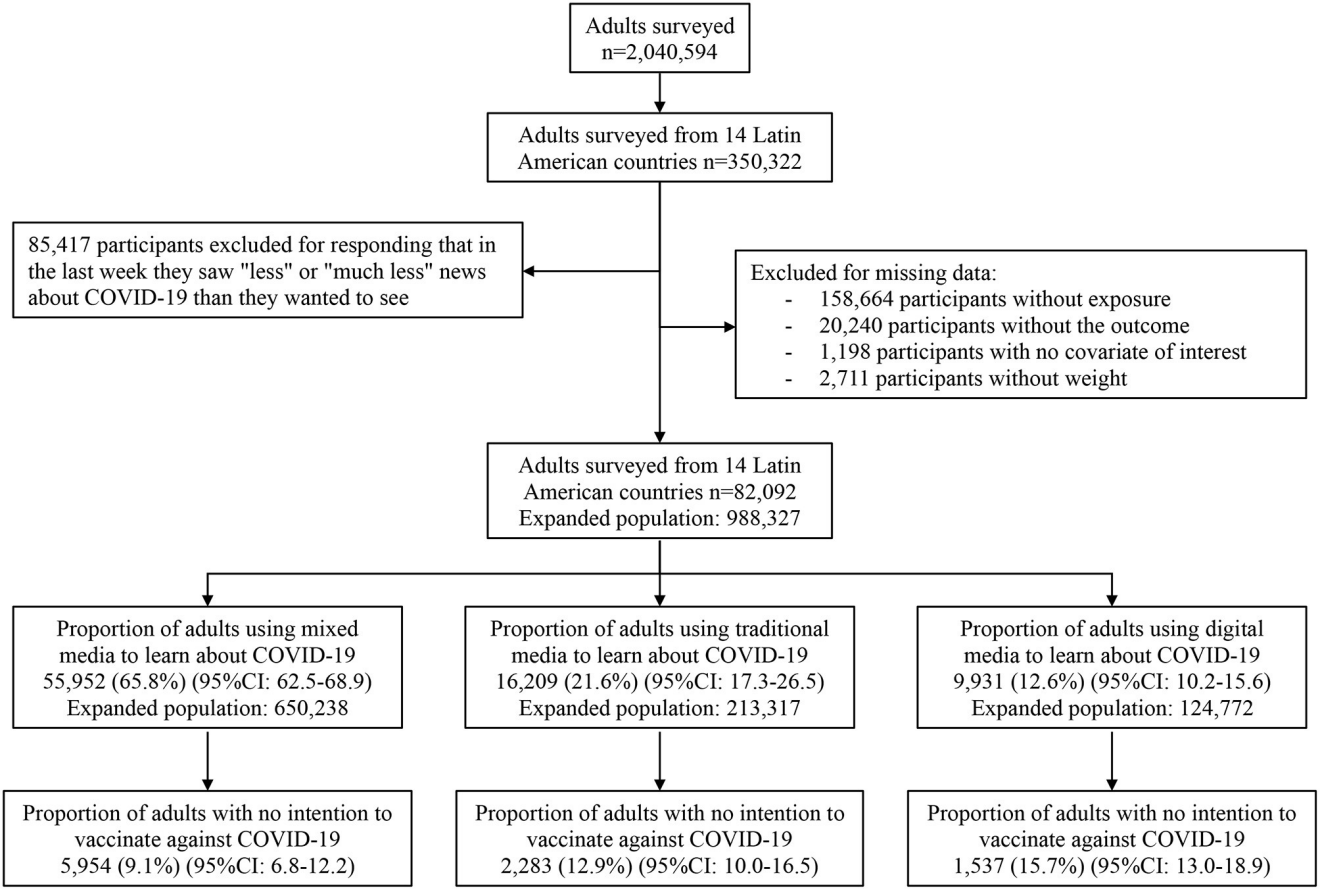
Flowchart of the selection of the study sample.

The population surveyed was 2,040,594 Facebook users over the age of 18 worldwide. For this study, participants residing in LAC countries (350,322), and those who in the past week saw about the right amount or more or much more news than they wanted to see about COVID-19 were included. All those who did not have information on the variables of interest were excluded. The final study population was 82,092 participants (Figure 1).

### Characteristics of the Study Samples

There was a higher proportion of men (50.0%), participants aged between 18 and 30 years (28.4%), with a secondary school education level (41.4%) and who lived in the city (86.1%). Likewise, the health condition reported in a greater proportion was good (34.4%). The prevalence of the use of the media to learn about COVID-19 was mostly through mixed media (65.8%). The non-intention of vaccination was 10.8% (Table 1).

**Table 1:** General characteristics of the study sample (*n* = 82,092; *N* = 988,327).

**Characteristics**	**Absolute frequency**	Weighted proportion*	
	***n***	**%**	**95%CI**
**Gender**			
Female	44,699	49.6	48.5-50.6
Male	37,211	50.0	48.8-51.2
Not binary	182	0.4	0.3-0.5
**Age (years)**			
18–30	24,489	28.4	24.0–33.2
31–40	19,879	20.5	19.2–22.0
41–50	15,872	18.4	17.7–19.1
51–60	13,002	16.5	15.6–17.4
61–70	6,839	11.9	10.5–13.4
71–80	1,774	3.7	2.8–4.7
80 or more	237	0.6	0.4–0.9
**Education level**			
Less than primary school	844	3.2	1.1–9.0
Primary school	4,553	9.0	5.2–15.3
Secondary school	30,494	41.4	35.7–47.4
College / University	35,851	35.4	22.8–50.5
Graduate school	10,350	10.9	8.9–13.3
**Area of residence**			
City	67,341	86.1	75.0–92.8
Town	9,776	8.9	3.3–21.6
Village or rural area	4,975	5.0	4.3–5.8
**Health condition**			
Poor	1,942	3.1	2.4–4.1
Fair	12,331	17.7	15.7–19.8
Good	27,575	34.4	31.5–37.4
Very good	25,680	28.0	26.5–29.6
Excellent	14,564	16.8	13.5–20.8
**Mass media used to learn about COVID-19**			
Mixed	55,952	65.8	62.5–68.9
Traditional	16,209	21.6	17.3–26.5
Digital	9,931	12.6	10.2–15.6
**Vaccination intention**			
Yes	72,318	89.2	86.4–91.5
No	9,774	10.8	8.5–13.6

*95%CI: 95% Confidence interval*.

* *Weights and the design effect of the complex survey sampling were included*.

### Proportion of Non-vaccination Intention According to the Mass Media Used to Learn About COVID-19 in Each LAC Country

**Figure 2:**
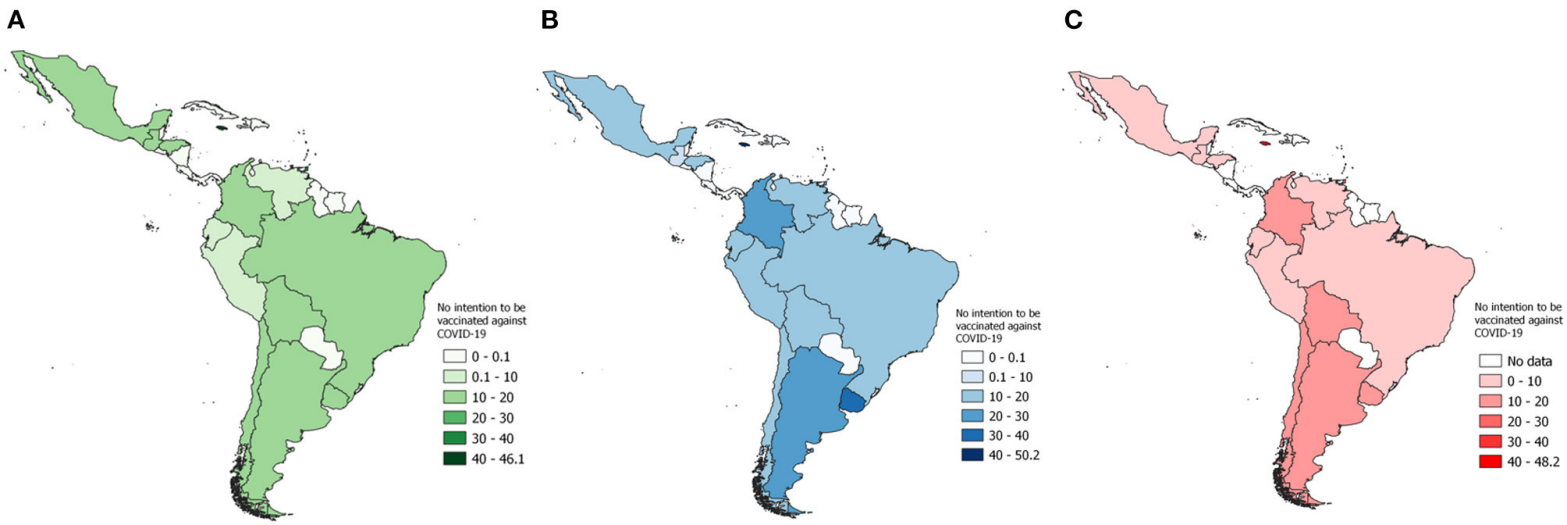
Proportion of non-vaccination intention according to the mass media used to learn about COVID-19 for each LAC country: **(A)** Traditional media; **(B)** Mixed media; **(C)** Digital media.

The proportion of non-vaccination intention according to the means of communication used to learn about COVID-19 varied by country in LAC. In relation to participants using traditional media, the proportion not intending to be vaccinated was highest in Jamaica (46.1%), Trinidad and Tobago (32.1%), and Uruguay (17.9%), while the lowest proportions were in Venezuela (8.0%), Ecuador (8.8%) and Peru (9.2%) (Figure 2A). In those using mixed media, the proportion not intending to be vaccinated was highest in Jamaica (48.2%), Trinidad and Tobago (26.5%), and Uruguay (19.6%), while the proportion was lowest in Brazil (7.1%), Ecuador (7.7%) and Peru (8.2%) (Figure 2B). In participants who used digital media, the proportion not intending to be vaccinated was highest in Jamaica (50.2%), Uruguay (37.5%), and Trinidad and Tobago (29.5%), and the lowest proportion was in Guatemala (7.8 %), Ecuador (13.3%) and Honduras (13.3%) (Figure 2C).

### Bivariate Analysis According to the Media Used to Learn About COVID-19

We found a higher proportion of non-vaccination intention among participants who used only traditional (12.9%) and digital (15.7%) media compared to those who used mixed media (9.1%) (*p* = 0.003). Likewise, we observed statistically significant differences according to sex, age, level of education, area of residence, and health status (Table 2).

**Table 2:** General characteristics according to each mass media group used in LAC (*n* = 82,092; *N* = 988,327).

**Characteristics**	**Mixed**		**Traditional**		**Digital**		***p*** -Value*
	***n***	**%**	***n***	**%**	***n***	**%**	
**Vaccination intention**							0.003**
Yes	49,998	90.9	13,926	87.1	8,394	84.3	
No	5,954	9.1	2,283	12.9	1,537	15.7	
**Gender**							0.021**
Female	30,680	50.4	8,831	49.2	5,188	45.9	
Male	25,165	49.4	7,334	49.7	4,712	53.8	
Non-binary	107	0.2	44	1.1	31	0.3	
**Age (years)**							0.002**
18–30	17,284	30.2	3,420	18.9	3,785	35.2	
31–40	13,896	21.4	3,414	17.8	2,569	20.8	
41–50	10,826	18.3	3,404	20.2	1,642	16.3	
51–60	8,518	15.8	3,281	20.4	1,203	13.6	
61–70	4,289	10.8	1,970	15.6	580	10.8	
71–80	1,012	3.2	622	5.5	140	3.1	
80 or more	127	0.3	98	1.6	12	0.2	
**Education level**							<0.001**
Less than primary school	444	2.6	306	5.0	94	2.9	
Primary school	2,333	7.3	1,845	14.9	375	8.2	
Secondary school	19,578	39.9	7,784	48.7	3,132	37.3	
College / University	25,731	38.0	5,114	24.6	5,006	40.3	
Graduate school	7,866	12.2	1,160	6.8	1,324	11.3	
**Living area**							0.030**
City	46,311	87.1	12,950	83.4	8,080	85.3	
Town	6,433	8.2	2,080	10.3	1,263	10.0	
Village or rural area	3,208	4.6	1,179	6.3	588	4.7	
**Health condition**							<0.001**
Poor	1,190	2.8	511	4.2	241	3.3	
Fair	8,022	16.8	2,831	20.7	1,478	16.7	
Good	19,024	35.1	5,404	33.2	3,147	32.6	
Very good	18,242	29.2	4,305	23.9	3,133	29.0	
Excellent	9,474	16.1	3,158	18.0	1,932	18.4	

*95%CI: 95% Confidence interval*.

*Weights and the design effect of the complex survey sampling were included*.

* *Refers to the statistical significance obtained from the comparison of the proportions between the categories of the variables considering the complex sampling of the survey*.

** *Pearson Chi-square test with Rao-Scott correction*.

### Bivariate Analysis According to the Non-intention of Vaccination Against COVID-19

The bivariate analysis according to the non-vaccination intention showed statistically significant differences for the means of communication used and for the health condition of the participants (Table 3).

**Table 3:** General characteristics according to vaccination intention in LAC (*n* = 82,092; *N* = 988,327).

**Characteristics**	**Vaccination intention**						
	**No**			**Yes**			***p*** -Value*
	***n***	**%**	**95%CI**	***n***	**%**	**95%CI**	
**Mass media used to learn about COVID-19**							0.003**
Mixed	5,954	9.1	6.8–12.2	49,998	90.9	87.8–93.2	
Traditional	2,283	12.9	10.0–16.5	13,926	87.1	83.5–89.9	
Digital	1,537	15.7	13.0–18.9	8,394	84.3	81.1–87.0	
**Gender**							0.860**
Female	5,851	10.9	7.6–15.4	38,848	89.1	84.6–92.4	
Male	3,881	10.6	9.3–12.1	33,330	89.4	87.9–90.7	
Non-binary	42	10.8	2.8–33.6	140	89.2	66.4–97.2	
**Age (years)**							0.144**
18–30	2,670	10.2	7.5–13.8	21,819	89.8	86.2–92.5	
31–40	2,380	11.3	8.6–14.7	17,499	88.7	85.3–91.4	
41–50	1,905	10.5	8.3–13.2	13,967	89.5	86.8–91.7	
51–60	1,652	10.5	7.9–14.0	11,350	89.5	86.0–92.1	
61–70	920	12.4	11.2–13.8	5,919	87.6	86.2–88.8	
71–80	223	10.5	8.5–12.8	1,551	89.5	87.2–91.5	
80 or more	24	5.1	2.0–12.4	213	94.9	87.6–98.0	
**Education level**							0.270**
Less than primary school	115	12.1	7.3–19.3	729	87.9	80.7–92.7	
Primary school	608	11.1	8.9–13.7	3,945	88.9	86.3–91.0	
Secondary school	3,894	11.2	8.6–14.4	26,600	88.8	85.6–91.4	
College / University	4,117	10.5	8.3–13.0	31,734	89.5	87.0–91.6	
Graduate school	1,040	9.8	7.2–13.1	9,310	90.2	86.8–92.8	
**Area of residence**							0.016**
City	7,298	10.4	8.3–13.1	60,043	89.6	86.9–91.7	
Town	1,484	12.7	9.6–16.6	8,292	87.3	83.4–90.4	
Village or rural area	992	14.0	12.2–16.1	3,983	86.0	83.9–87.8	
**Health condition**							<0.001**
Poor	227	11.6	9.8–14.7	1,715	88.4	85.3–90.2	
Fair	1,206	9.0	7.7–10.6	11,125	91.0	89.4–92.3	
Good	2,701	8.4	6.3–11.1	24,874	91.6	88.9–93.7	
Very good	3,073	10.6	8.1–13.8	22,607	89.4	86.2–91.9	
Excellent	2,567	17.7	15.2–20.5	11,997	82.3	79.5–84.8	

*95%CI: 95% Confidence interval*.

*Weights and the design effect of the complex survey sampling were included*.

* *Refers to the statistical significance obtained from the comparison of the proportions between the categories of the variables considering the complex sampling of the survey*.

** *Pearson Chi-square test with Rao-Scott correction*.

### Association Between the Mass Media Used to Learn About COVID-19 and the Non-intention of Vaccination Against COVID-19

In the crude analysis, a higher prevalence of not intending to be vaccinated against COVID-19 was found in those who used traditional media (cPR = 1.42; 95%CI: 1.35–1.49; *p* < 0.001) and digital media (cPR = 1.72; 95%CI: 1.21–2.46; *p* = 0.006) compared to those using mixed media. This association remained statistically significant in the analysis adjusted for sex, age, education level, living area and health condition [traditional media (aPR = 1.36; 95%CI: 1.29–1.44; *p* < 0.001); digital media (aPR = 1.70; 95%CI: 1.24–2.33; *p* = 0.003)] (Table 4).

**Table 4:** Crude and adjusted prevalence ratio for non-intention to vaccinate according to each mass media group used to learn about COVID-19.

**No vaccination intention**	**Crude Model^a^**		**Adjusted Model^a,b^**	
	**cPR (95%CI)**	***p*** **-Value**	**aPR (95%CI)**	***p*** **-Value**
**Mass media used**				
Mixed	Ref.	—	Ref.	—
Traditional	1.42 (1.35–1.49)	<0.001	1.36 (1.29–1.44)	<0.001
Digital	1.72 (1.21–2.46)	0.006	1.70 (1.24–2.33)	0.003

*cPR: crude prevalence ratio; aPR: adjusted prevalence ratio; 95%CI: 95% Confidence interval*.

a *A generalized linear model of the Poisson family was carried out with link log considering the effect of the design and the weights of the complex sampling of the survey*.

b *Adjusted for sex, age, education level, living area and health condition*.

## Discussion

The objective of this study was to evaluate the association between the use of mass media news and information to learn about COVID-19 and the non-intention of vaccination against COVID-19. As a result, it was found that people who use only traditional or digital media had a higher non-vaccination intention compared to those who use both types of information about COVID-19.

The COVID-19 pandemic is a scenario of health challenges due to the so-called infodemic (27), and therefore, it is important that accessible information on health issues is reliable (28). Some studies indicate that mass media news and information could be an important source of both information and disinformation about COVID-19. In Italy, a study showed that during the first wave of COVID-19, the Italian press preferred to resort to infodemic and moderately infodemic terms, while scientific sources favored the correct names (29). In Brazil, a study found that a social network such as Twitter had coverage of topics related to COVID-19 similar to that of the media (25). In addition, some media reports presented a negative feeling toward political issues in the media and that a high incidence of mentions of a specific drug denoted a high political polarization during the pandemic (25). Another investigation showed that between May and June 2020, the top six terms related to COVID-19 searched on Google were “coronavirus,” “corona,” “COVID,” “virus,” “corona virus” and “COVID-19” (30). The countries with a higher number of COVID-19 cases had a higher number of COVID-19 queries on Google. Searches for “tips and cures” for COVID-19 increased in connection with the then-president of the United States speculating about a “miracle cure” and suggesting an injection of disinfectant to treat the virus (30). This same study noted that around two-thirds of Instagram users used the hashtags “COVID-19” and “coronavirus” to spread information related to the virus (30).

In relation to the intention to vaccinate against COVID-19, it has been described that exposure to misinformation leads to less intention to vaccinate, including people who, before being exposed to misinformation, indicated that they would definitely get vaccinated (31). It has also be described that exposure to misinformation about COVID-19 decreases the intention to vaccinate in those people who are motivated to get vaccinated to provide protection against the disease to family members, friends or other people at risk (31).

Several studies have described the influence of the mass media news and information on the intention of vaccination against COVID-19, with results that disagree with what we found. One study showed that newspapers could be a source of information that increases vaccination intention (32). Other research showed that in the United States population, traditional media such as both local and national television and national newspapers are used more to obtain information about the COVID-19 vaccine and that these means increase the probability of vaccination (33). On the other hand, another study also carried out in the United States found that compared with people who use digital media, those who use traditional media to know about COVID-19 had a greater intention to vaccinate (33). It is likely that these discrepancies are due to the quality of the information of the traditional media in LAC and the distrust that people who seek more reliable information have about them (34–36). This can lead to the consumption of these media by people who are more likely to accept information that encourages their own misconceptions about the vaccine, as in Italy, where the press gave false news (29). Given the spread of false and erroneous information about post-immunization deaths at the beginning of the vaccination campaign in Italy, it was reported that between 10 and 20% of Italian candidates for the AstraZeneca vaccine rejected it, causing a delay in vaccination and the non-administration of ~200,000 doses (37).

The effect of digital media on non-vaccination intention has been previously described. In England, a study associated vaccine reluctance with belief in conspiracy theories and attitudes in general toward vaccines, as well as an informative dependency on social networks (38). In the United States, it was found that persons who are less likely to receive the COVID-19 vaccine, use social networks as their only source of information, or at least as one of their sources of information (33, 39). This preponderance of digital media among people who do not have the intention of vaccination may be due to the influence of the anti-vaccine movement in these media. Indeed, previous research has identified social networks such as Facebook and Twitter as popular platforms for members of the anti-vaccine movement (40). In recent years, this movement has expanded to all major digital platforms, including YouTube, Instagram, and personal messaging services such as WhatsApp, and during the pandemic, the growth of this movement accelerated (41).

Although our study did not evaluate the possible reasons, it is likely that the greater intention to vaccinate against COVID-19 in people who seek information in both traditional and digital media is due to the fact that they are people who contrast information. In other words, before accepting information from a single medium, they decide to compare its veracity with other media. This form of information would make them less susceptible to misinformation and, therefore, more likely to accept vaccination.

The media are essential for the public to acquire scientific information from reliable, authoritative, and responsible sources, and people can even use these sources when they want to convince others (41). This poses some challenges that governments must face to improve their strategies of communication, such as the monitoring of social networks for timely changes in strategies or even the design of specific communication strategies to modify the intention of vaccination. One study identified top themes related to COVID-19 vaccines in tweets globally. The tweets were related to negative sentiments and largely framed the themes of emotional reactions and public concerns related to COVID-19 vaccines (42). Tweets related to facilitators of vaccination showed temporal variations over time, while barrier-related tweets remained largely constant throughout the study period (42). A study in Pakistan explored the potential effects of various communication strategies and identified fear appraisal as the most viable communication strategy for combating vaccine hesitancy (43). In addition, public skepticism negatively moderated the effects of media types and attributes of public service messages on willingness to get vaccinated (43). These strategies should consider the credibility of each of the media to employ. For example, in Germany, a study examined the relationship between exposure and credibility of different sources of health information and vaccination intention against COVID-19. The results revealed that in addition to reliable information from experts and health authorities, local newspapers also have a positive impact on vaccination intention (32). However, this effect decreases to a certain extent when age is considered. Furthermore, alternative information sources pose a notable threat to vaccination intent against COVID-19 (32). In the context of vaccination against COVID-19, a study evaluated that mechanisms such as the perception of information and persuasion of the individual affect attitudes toward vaccination (44). This is based on the influence of the completeness of the information, the veracity of the information, as well as the exchange of experiences and social pressure on the individual attitude of people, representing important aspects in the dissemination of information (44).

Our study has some limitations. The cross-sectional design does not allow causal relationships to be established between the variables of interest. Likewise, the universe studied corresponds to the population that has a social network, that is, the population with Internet access, reducing the generalization of results to the population that does not have this access. Additionally, some variables that would have made it possible to better characterize the phenomenon under study were not included in the analysis since the inclusion of variables was dependent on their availability in the database. It should also be mentioned that the survey data were obtained by self-reporting, and thus, memory or social desirability bias could occur. Although these limitations affect the generalization of the results obtained for the general population of LAC, the use of the social network Facebook in this region of the world is high, with four out of five inhabitants of this region using Facebook, thereby making these data useful for an initial approach to study the relationship between the sources of news and information on COVID-19 and the intention to vaccinate against this disease.

In conclusion, it was found that in the LAC population, two out of 10 people who only used digital media to learn about COVID-19 had no intention of vaccination, while only one in 10 people who used traditional or traditional and digital media had no intention of vaccination. We found an association between the type of information source used to learn about COVID-19 and the non-intention of vaccination. The use of only traditional or digital information sources was associated with a higher probability of non-intention of vaccination compared to the use of both information sources. Given this scenario, the communication of information supported by scientific evidence should be promoted as well as the development of strategies aimed at promoting vaccination in populations with less intention of receiving the COVID-19 vaccine. Reporting by traditional media and social media companies should be aimed at addressing vaccine hesitancy and the dissemination of correct and easy-to-digest information to the public.

## Data Availability Statement

The datasets analyzed in this research article are not publicly available. This datasets were shared by the Massachusetts Institute of Technology due to signed agreement. Any request of them should be directed to vbeniteszapata@gmail.com.

## Ethics Statement

Ethical review and approval was not required for the study on human participants in accordance with the local legislation and institutional requirements. The patients/participants provided their written informed consent to participate in this study.

## Author Contributions

GB-Q, JB-M, DU-P, and VB-Z participated in concept design and supervising the study. PH-A, AU-C, AR-M, CT-H, and AH participated in concept design. JB-M, DU-P, and VB-Z conducted the statistical analysis. All authors participated in manuscript writing, editing, final revision, and have read and agreed on the submitted manuscript.

## Conflict of Interest

The authors declare that the research was conducted in the absence of any commercial or financial relationships that could be construed as a potential conflict of interest.

## Publisher's Note

All claims expressed in this article are solely those of the authors and do not necessarily represent those of their affiliated organizations, or those of the publisher, the editors and the reviewers. Any product that may be evaluated in this article, or claim that may be made by its manufacturer, is not guaranteed or endorsed by the publisher.
