# Supplementary material for: Mass Media Use to Learn About COVID-19 and the Non-intention to Be Vaccinated Against COVID-19 in Latin America and Caribbean Countries
Source: Front Med (Lausanne). 2022 Jun 13;9:877764. doi: 10.3389/fmed.2022.877764 (PMC9234268; doi:10.3389/fmed.2022.877764)
Supplement: Supplementary file 1 [file Table_1.DOCX]

**Supplementary material**

| **Table S1.** **Proportion of vaccination intention among subjects who used mixed media to learn about COVID-19 in each LAC country.** | | | | | |
| --- | --- | --- | --- | --- | --- |
| **Countries** | **Vaccination intention** | | | | |
|  |  | **Yes** | | **No** | |
|  |  | **Weighted proportion** | | **Weighted proportion** | |
|  | **N** | **%** | **95%CI** | **%** | **95%CI** |
| Argentina | 14985 | 86.6 | 85.7-87.4 | 13.4 | 12.6-14.3 |
| Bolivia | 2334 | 89.8 | 87.0-92.1 | 10.2 | 7.9-13.0 |
| Brazil | 15910 | 92.9 | 92.2-93.5 | 7.1 | 6.5-7.8 |
| Chile | 2068 | 83.0 | 80.3-85.3 | 17.0 | 14.7-19.7 |
| Colombia | 14525 | 86.1 | 85.2-87.0 | 13.9 | 13.0-14.8 |
| Ecuador | 2378 | 92.3 | 90.3-93.9 | 7.7 | 6.1-9.7 |
| Guatemala | 2421 | 90.2 | 87.6-92.2 | 9.8 | 7.8-12.4 |
| Honduras | 2049 | 91.6 | 89.4-93.4 | 8.4 | 6.6-10.6 |
| Jamaica | 1430 | 51.8 | 48.1-55.4 | 48.2 | 44.6-51.9 |
| Mexico | 15398 | 90.5 | 89.6-91.3 | 9.5 | 8.7-10.4 |
| Peru | 2398 | 91.8 | 89.4-93.8 | 8.2 | 6.2-10.6 |
| Trinidad & Tobago | 2021 | 73.5 | 70.1-76.6 | 26.5 | 23.4-29.9 |
| Uruguay | 2267 | 80.4 | 77.9-82.7 | 19.6 | 17.3-22.1 |
| Venezuela | 3066 | 91.3 | 89.4-92.8 | 8.7 | 7.2-10.6 |
| **95%CI:** 95% Confidence interval | | | | | |

| **Table S2.** **Proportion of vaccination intention among subjects who used traditional media to learn about COVID-19 in each LAC country.** | | | | | |
| --- | --- | --- | --- | --- | --- |
| **Countries** | **Vaccination intention** | | | | |
|  |  | **Yes** | | **No** | |
|  |  | **Weighted proportion** | | **Weighted proportion** | |
|  | **N** | **%** | **95%CI** | **%** | **95%CI** |
| Argentina | 14985 | 83.3 | 81.6-84.8 | 16.7 | 15.2-18.4 |
| Bolivia | 2334 | 89.6 | 85.6-92.6 | 10.4 | 7.4-14.4 |
| Brazil | 15910 | 89.4 | 87.6-91.0 | 10.6 | 9.0-12.4 |
| Chile | 2068 | 82.7 | 78.2-86.5 | 17.3 | 13.5-21.8 |
| Colombia | 14525 | 83.0 | 81.4-84.6 | 17.0 | 15.4-18.6 |
| Ecuador | 2378 | 91.2 | 87.2-94.0 | 8.8 | 6.0-12.8 |
| Guatemala | 2421 | 87.6 | 83.2-91.0 | 12.4 | 9.0-16.8 |
| Honduras | 2049 | 87.0 | 82.6-90.4 | 13.0 | 9.6-17.4 |
| Jamaica | 1430 | 53.9 | 45.7-61.8 | 46.1 | 38.2-54.3 |
| Mexico | 15398 | 86.5 | 84.4-88.5 | 13.5 | 11.5-15.6 |
| Peru | 2398 | 90.8 | 87.4-93.3 | 9.2 | 6.7-12.6 |
| Trinidad & Tobago | 2021 | 67.9 | 60.7-74.4 | 32.1 | 25.6-39.3 |
| Uruguay | 2267 | 82.1 | 78.5-85.2 | 17.9 | 14.8-21.5 |
| Venezuela | 3066 | 92.0 | 88.6-94.5 | 8.0 | 5.5-11.4 |
| **95%CI:** 95% Confidence interval | | | | | |

| **Table S3.** **Proportion of vaccination intention among subjects who used digital media to learn about COVID-19 in each LAC country.** | | | | | |
| --- | --- | --- | --- | --- | --- |
| **Countries** | **Vaccination intention** | | | | |
|  |  | **Yes** | | **No** | |
|  |  | **Weighted proportion** | | **Weighted proportion** | |
|  | **N** | **%** | **95%CI** | **%** | **95%CI** |
| Argentina | 14985 | 75.5 | 70.3-80.0 | 24.5 | 20.0-29.7 |
| Bolivia | 2334 | 85.4 | 77.8-90.7 | 14.6 | 9.3-22.2 |
| Brazil | 15910 | 83.3 | 80.7-85.6 | 16.7 | 14.4-19.3 |
| Chile | 2068 | 80.9 | 73.2-86.7 | 19.1 | 13.3-26.8 |
| Colombia | 14525 | 79.1 | 75.7-82.2 | 20.9 | 17.8-24.3 |
| Ecuador | 2378 | 86.7 | 81.2-90.8 | 13.3 | 9.2-18.8 |
| Guatemala | 2421 | 92.2 | 87.3-95.3 | 7.8 | 4.7-12.7 |
| Honduras | 2049 | 86.7 | 79.5-91.6 | 13.3 | 8.4-20.5 |
| Jamaica | 1430 | 49.8 | 40.9-58.8 | 50.2 | 41.2-59.1 |
| Mexico | 15398 | 87.4 | 85.5-89.0 | 12.6 | 11.0-14.5 |
| Peru | 2398 | 84.2 | 77.3-89.3 | 15.8 | 10.7-22.7 |
| Trinidad & Tobago | 2021 | 70.5 | 63.8-76.4 | 29.5 | 23.6-36.2 |
| Uruguay | 2267 | 62.5 | 53.0-71.1 | 37.5 | 28.9-47.0 |
| Venezuela | 3066 | 86.5 | 83.0-89.4 | 13.5 | 10.6-17.0 |
| **95%CI:** 95% Confidence interval. | | | | | |
